# Comparing ChatGPT Feedback and Peer Feedback in Shaping Students’ Evaluative Judgement of Statistical Analysis: A Case Study

**DOI:** 10.3390/bs15070884

**Published:** 2025-06-28

**Authors:** Xiao Xie, Lawrence Jun Zhang, Aaron J. Wilson

**Affiliations:** Faculty of Arts and Education, University of Auckland, Auckland 1010, New Zealand; orlandoxxie@gmail.com (X.X.); aj.wilson@auckland.ac.nz (A.J.W.)

**Keywords:** ChatGPT feedback, evaluative judgement, Higher Degree by Research students, peer feedback, statistical analysis

## Abstract

Higher Degree by Research (HDR) students in language and education disciplines, particularly those enrolled in thesis-only programmes, are increasingly expected to interpret complex statistical data. However, many lack the analytical skills required for independent statistical analysis, posing challenges to their research competence. This study investigated the pedagogical potential of ChatGPT-4o feedback and peer feedback in supporting students’ evaluative judgement during a 14-week doctoral-level statistical analysis course at a research-intensive university. Thirty-two doctoral students were assigned to receive either ChatGPT feedback or peer feedback on a mid-term assignment. They were then required to complete written reflections. Follow-up interviews with six selected participants revealed that each feedback modality influenced their evaluative judgement differently across three dimensions: hard (accuracy-based), soft (value-based), and dynamic (process-based). While ChatGPT provided timely and detailed guidance, it offered limited support for students’ confidence in verifying accuracy. Peer feedback promoted critical reflection and collaboration but varied in quality. We therefore argue that strategically combining ChatGPT feedback and peer feedback may better support novice researchers in developing statistical competence in hybrid human–AI learning environments.

## 1. Introduction

In the evolving landscape of educational research, Higher Degree by Research (HDR) students in language and education disciplines are increasingly expected to engage with empirical studies involving complex statistical analyses (Brown, 2014, 2024). The demand is particularly pressing for students enrolled in thesis-only programmes, where independent research constitutes the sole basis for academic progression. These students must not only engage meaningfully with the quantitative literature but also interpret statistical outputs accurately and assess methodological rigour critically (Albers, 2017; Baglin et al., 2017). The urgency of addressing research training gaps in HDR programmes cannot be overstated. A significant concern is that many HDR students are inadequately prepared to meet the complex demands of contemporary research. As Brown (2014, p. 70) observes, most students “have had little or no undergraduate preparation in research design or data analysis (quantitative or qualitative) and lack knowledge of the epistemological foundations for social science research”. This issue is especially acute in areas requiring quantitative analysis, where students often enter their programmes with minimal formal training in statistics or research design (Albers, 2017; Baglin et al., 2017). Consequently, they are frequently ill-equipped to undertake rigorous empirical inquiry. Many struggle to grasp core statistical concepts, apply appropriate analytical techniques, and, crucially, make informed judgements about the quality and implications of statistical findings (Brown, 2024). This analytical gap not only compromises their ability to conduct robust research but also limits their participation in scholarly discourse, which increasingly demands data-literate engagement (Baglin et al., 2017).

Addressing this gap requires more than the transmission of statistical knowledge; it necessitates the cultivation of evaluative judgement, namely, “to make decisions about the quality of work of oneself and others” (Tai et al., 2018, p. 467). Evaluative judgement is a vital metacognitive capacity in research-intensive education, encompassing the ability to distinguish between high- and low-quality work, recognise methodological limitations, and justify research decisions. For HDR students, this involves evaluating the accuracy, relevance, and integrity of statistical analyses not only in published research but also in their own work and that of their peers. Cultivating this capacity is central to fostering academic autonomy and critical scholarship, supporting students to engage in methodologically sound and intellectually rigorous research practices. Crucially, the significance of evaluative judgement extends beyond discrete tasks or assessments; it underpins the broader goal of preparing students for lifelong learning and reflective professional practice. This aligns with Boud’s (2000) concept of sustainable assessment, which emphasises curriculum design that equips learners to engage with and navigate standards of quality across future learning contexts (Boud & Soler, 2016).

In response to these challenges and opportunities, recent scholarship has increasingly positioned feedback as a central pedagogical strategy for cultivating evaluative judgement (Bearman et al., 2024; Tai et al., 2016, 2018; Zhan et al., 2025). Two distinct yet potentially complementary feedback mechanisms have emerged: peer feedback and artificial intelligence (AI)-generated feedback that uses tools such as ChatGPT.

Peer feedback is widely recognised for its capacity to foster critical thinking and reflective learning through interpersonal dialogue. Engaging in mutual evaluation enables students to articulate assessment criteria, assess the quality of work, and justify their judgements, which are key components in the development of evaluative judgement (Tai et al., 2016). To further enhance this capacity, research suggests that students should be given more structured opportunities to observe and evaluate their peers’ work (Chen et al., 2022). When combined with a dialogic approach to feedback, such experiences can significantly enhance the development of evaluative judgement (Tai et al., 2018). Crucially, it is important that structured scaffolding be the approach using strategies such as resorting to explicit evaluation criteria, expert facilitation, and strong disciplinary framing to ensure that peer feedback serves as a constructive and intellectually rigorous learning mechanism (Xie et al., 2024a, 2024b).

In contrast to traditional forms of feedback, the rapid advancement of generative AI presents both promising opportunities and complex challenges for the development of evaluative judgement. As these tools become increasingly integrated into academic and research environments, students are not only expected to know how to use generative AI but also to engage critically with the outputs it produces and the processes that underpin its functioning (Huang et al., 2023). Tools like ChatGPT, for example, offer immediate, personalised feedback, thereby expanding access to formative support, especially in settings where instructional resources are limited. AI tools’ ability to provide consistent, on-demand assistance offers a scalable solution for supporting independent inquiry and iterative learning. Yet, this technological affordance alone is insufficient. As Markauskaite et al. (2022) argue, preparing learners for a world with AI requires more than technical proficiency; it demands the cultivation of cognitive, humanistic, and social capacities that enable individuals to navigate the ethical, epistemic, and collaborative dimensions of AI-mediated knowledge practices. These overlapping perspectives shift the educational focus from understanding AI as a tool to recognising the cognitive agility, value orientation, and collective reasoning required to operate effectively in AI-infused contexts. Within this evolving landscape, Bearman et al. (2024) offer a compelling framework for understanding how evaluative judgement intersects with generative AI across three dimensions: “(1) developing evaluative judgement of generative AI outputs; (2) developing evaluative judgement of generative AI processes; and (3) generative AI assessment of student evaluative judgements” (p. 893). This framework underscores the need for students to critically assess not only what AI produces but how it produces it and to reflect on how AI may, in turn, assess their own reasoning and decisions. Such engagement transforms evaluative judgement from a static academic skill into a dynamic, reflexive practice, one that is essential for navigating the opportunities and ambiguities of learning and researching with AI.

While ChatGPT holds promise for enhancing statistical education, particularly through its ability to generate codes, explain procedures, and support learners with limited backgrounds (Ellis & Slade, 2023; Schwarz, 2025), its limitations warrant careful consideration. Despite its vast training on statistical content and the data science literature, ChatGPT lacks true interpretive understanding and cannot independently validate claims or assess methodological appropriateness (Xing, 2024). This becomes particularly problematic in statistical analysis, where AI has been found to produce conceptual inaccuracies, misguide procedural choices, and occasionally offer flawed software instructions. A key concern is the risk of ‘‘hallucination’, the confident generation of incorrect or fabricated information when the AI lacks sufficient training data (Rudolph et al., 2023). Moreover, ChatGPT’s reliance on historical data restricts its capacity for innovation or original reasoning, and its lack of personal experience limits context-sensitive judgement. These shortcomings underscore the importance of human oversight and disciplinary expertise. As Xing (2024) notes, the pedagogical value of AI feedback depends not on output fluency, but on students’ critical capacity to assess its validity and relevance. Thus, ChatGPT should not be viewed as a flawless authority, but rather as a hybrid educational actor: a knowledge-rich assistant to be supervised, a peer that prompts critical thinking, a student whose errors we must identify, and a teacher from whom broad understanding, not definitive answers, can be drawn.

Despite growing interest in these feedback modalities, their pedagogical implications remain underexplored, particularly in the context of quantitative research training for HDR students in non-STEM (Science, Technology, Engineering, and Mathematics) disciplines. Empirical evidence is still limited regarding how different feedback mechanisms influence the development of evaluative judgement when students work with complex statistical content. To address this gap, the present study investigated how peer feedback and ChatGPT feedback would shape HDR students’ evaluative judgement within a 14-week doctoral-level statistical analysis course at a research-intensive university. Drawing on students’ written reflections and semi-structured interviews, this study intends to contribute to emerging understandings of how feedback operates in hybrid human–AI learning environments. It also offers practical guidance for designing postgraduate education that supports students’ critical and autonomous engagement with statistical reasoning in contemporary educational research.

## 2. Literature Review

### 2.1. Defining Evaluative Judgement

The conceptualisation of evaluative judgement has undergone significant refinement in recent years, accompanied by increasing scholarly interest in its theoretical foundations and pedagogical relevance to higher education (Ajjawi et al., 2018). A foundational contribution by Tai et al. (2016) defines evaluative judgement as the capacity to both discern what constitutes quality and apply this understanding when assessing one’s own work or that of others. While this definition has gained prominence in contemporary educational discourse, its origins can be traced back to Sadler’s (1989) formative assessment theory, which emphasised the development of evaluative knowledge through sustained engagement with exemplars, explicit criteria, and feedback. Sadler’s work laid the groundwork for later conceptual developments by virtue of its foregrounding the importance of enabling students to make informed judgements about the quality of academic performance.

Subsequent research has enriched the theoretical landscape of evaluative judgement by introducing a range of conceptual lenses that highlight its multifaceted nature, as demonstrated in Table 1. Ajjawi and Bearman (2018), adopting a sociomaterial perspective, conceptualise evaluative judgement as emerging through the dynamic interplay between learners, tools, and contextual factors. In parallel, Dall’Alba (2018) situates evaluative judgement within both the epistemological dimension, what students know and can do, and the ontological dimensions, who they are becoming in the digitally mediated context of higher education. Joughin (2018) contributes to this discourse by applying dual-process theory, which distinguishes between two modes of reasoning involved in judgement: an intuitive, automatic, and unconscious process, and a deliberate, analytical, and effortful one. Adding further nuance, Goodyear and Markauskaite (2018) frame evaluative judgement as an epistemic capability, one that supports knowledgeable action in complex and evolving contexts. Building on these foundations, Luo and Chan (2022a, 2022b) reconceptualise evaluative judgement as a developmental and integrative construct, encompassing cognitive, affective, behavioural, and identity-related dimensions. Rather than viewing it as a discrete skill, they argue that evaluative judgement is a holistic capability that evolves through sustained engagement in disciplinary practices.

A particularly salient contribution to the current study is Nelson’s (2018) tripartite framework, which offers a historically grounded typology of evaluative judgement. Drawing on disciplinary and temporal shifts in assessment practices, Nelson distinguishes between three interrelated dimensions of evaluative judgement: hard, soft, and dynamic. This classification provides a nuanced lens for understanding how students engage in evaluative reasoning, not only in terms of assessing accuracy but also considering significance or value and adapting judgements through ongoing procedural reflection. As such, the framework captures the multifaceted nature of evaluative judgement as it unfolds across diverse educational contexts. Hard evaluative judgement involves the objective assessment of correctness, typically grounded in paradigmatic standards such as grammatical accuracy or technical precision. Soft evaluative judgement, by contrast, entails a subjective appraisal of the quality or significance of work, focusing less on right-or-wrong dichotomies and more on interpretive judgements of value. Dynamic evaluative judgement captures the procedural and communicative aspects of evaluative reasoning, namely, how ideas are organised, structured, and conveyed in ways that engage an audience. Crucially, evaluative judgement is not a universally transferable skill but is shaped by domain-specific standards and disciplinary conventions. Expertise in a given field enables individuals to make informed assessments of quality within that context.

Applying Nelson’s (2018) framework, Chong (2021) conducted an empirical study on IELTS writing preparation using exemplars. Chong illustrated hard evaluative judgement through grammatical and mechanical accuracy (e.g., correct verb tenses and punctuation), soft evaluative judgement through lexical and syntactic variety, and dynamic evaluative judgement through the logical structuring of ideas to communicate effectively with readers. Building on this work, Xie et al. (2024a) examined how different peer feedback roles influence the development of evaluative judgement in a tertiary English argumentative writing course in Malaysia. Twenty-four undergraduates were randomly assigned to act as feedback providers, receivers, or outsiders during structured peer feedback sessions. A thematic analysis of pre- and post-intervention surveys revealed that these roles differentially supported the cultivation of the three types of evaluative judgement. Although students’ domain-specific expertise was limited, the purposeful integration of peer feedback into writing instruction nonetheless created opportunities for developing nuanced judgement across hard, soft, and dynamic dimensions.

While Nelson’s (2018) tripartite framework of evaluative judgement, comprising hard, soft, and dynamic dimensions, has demonstrated substantial value in writing-focused contexts, its applicability to other disciplinary domains, such as statistical data analysis, remains insufficiently explored. Given the distinct epistemic practices, reasoning modalities, and evaluative standards that underpin statistical analysis, it is plausible that the manifestations of evaluative judgement in this domain diverge from those observed in more interpretive or humanities-based settings. This disciplinary specificity underscores the need for conceptual refinement and contextual adaptation to ensure the framework’s relevance and analytic precision. To address this gap, the present study adopts Nelson’s (2018) model as a theoretically grounded yet flexible heuristic to investigate the development of HDR students’ evaluative judgement as they engage with different feedback modalities (e.g., peer feedback or AI-generated feedback) in a doctoral-level statistics course.

Nelson’s (2018) typology provides a robust integrative scaffold that enables this study to synthesise and differentiate insights from a diverse range of theoretical perspectives. For example, hard evaluative judgement, which focuses on the assessment of correctness and procedural accuracy, aligns closely with Joughin’s (2018) dual-process theory, particularly its emphasis on deliberate, analytical reasoning (System 2). In contrast, soft evaluative judgement, which concerns the appraisal of values and significance, resonates with Dall’Alba’s (2018) ontological framing, which highlights the role of epistemological becoming within disciplinary communities. Meanwhile, dynamic evaluative judgement, which captures the procedural and communicative organisation of evaluative reasoning, shares theoretical ground with Luo and Chan’s (2022a, 2022b) developmental perspective that foregrounds the interplay of identity, cognition, and engagement in disciplinary practices, emphasising the holistic, non-linear development of evaluative judgement over time. Notably, Nelson’s (2018) framework implicitly echoes this latter view by recognising the disciplinary embeddedness of evaluative criteria and reasoning processes. In light of these complementarities and theoretical tensions, Nelson’s (2018) tripartite model offers both conceptual breadth and analytical clarity. Its dimensional structure facilitates a nuanced investigation into how HDR students interpret and respond to feedback, refine their understanding of quality in statistical analysis, and gradually develop the capacity to exercise evaluative judgement in increasingly autonomous and discipline-specific ways. By applying Nelson’s (2018) framework to a quantitatively oriented educational context, this study not only extends the empirical application of the model but also contributes to its theoretical development, illustrating its value in capturing the complex, situated, and evolving nature of evaluative judgement across disciplinary domains.

### 2.2. Developing Evaluative Judgement of Statistical Analysis

In today’s data-intensive research environment, doctoral and HDR students in language and education disciplines are increasingly expected to master complex quantitative methodologies. These advanced skills are not only vital for conducting rigorous empirical studies but also for critically engaging with statistical findings in the academic literature. As Brown (2024) notes, these methodologies include “psychometric test analysis, structural equation modelling, hierarchical level modelling, missing value analysis, or propensity score analysis” (p. 1).

Despite the increasing emphasis on quantitative competence, many doctoral students in language and education continue to face a substantial gap in the statistical skills required for advanced research. Most enter HDR programmes with minimal exposure to statistical reasoning, probability theory, or data analysis. Their prior education typically emphasises relational and pedagogical capacities, such as curriculum design, instructional practice, pastoral care, and formative assessment, rather than technical expertise in research methodology. This lack of preparation makes it difficult for students to navigate quantitative research with confidence, limiting their ability to engage meaningfully in data-informed scholarship and to function as autonomous researchers. Brown (2024) highlights this concern, pointing out that students often struggle to interpret or apply quantitative methods effectively. Empirical evidence further substantiates this gap. Baglin et al. (2017), in a survey of 191 research supervisors at an Australian university, reported a marked discrepancy between students’ actual and required statistical knowledge. While 76.6% of supervisors rated students’ statistical proficiency as introductory or lower, 74.5% indicated that at least an intermediate level was necessary to complete their research degrees successfully. This clear disconnect signals an urgent need to reform doctoral-level research training in education to better align with the analytical demands of contemporary academic inquiry.

A central challenge in addressing this issue is designing research training that not only imparts foundational statistical knowledge but also cultivates the critical thinking skills required to apply it meaningfully. Teaching quantitative data analysis should move beyond procedural instruction and rote calculation; it must be approached as a mode of reasoning that enables students to explore patterns, identify relationships, and make valid inferences grounded in data. The goal is not simply to teach statistical tests, but to enable students to use those tests as tools for drawing sound, contextually relevant conclusions. As Albers (2017) explains, three pedagogical goals are essential for fostering meaningful engagement with quantitative analysis: “(a) determining what questions to ask during all phases of a data analysis, (b) recognising how to judge the relevance of potential questions, and (c) deciding how to understand the deep-level relationships within the data” (p. 215).

In response to this challenge, the present study builds upon Nelson’s (2018) tripartite typology by redefining hard, soft, and dynamic evaluative judgement within the specific context of statistical data analysis. These adapted definitions aim to capture the nuanced ways in which students engage with the accuracy, relevance, and procedural quality of statistical tasks.

Hard evaluative judgement refers to the critical, accuracy-oriented appraisal of statistical outputs. This involves identifying errors, assessing the appropriateness of chosen methods, and verifying procedural correctness to ensure that results are both methodologically sound and factually accurate. It is a rigorous process grounded in standards and evidence-based reasoning, distinguishing correct from incorrect interpretations. Without hard evaluative judgement, both AI-supported and peer-mediated analyses may overlook methodological flaws, thereby compromising the validity and credibility of research findings.

Soft evaluative judgement, by contrast, involves a reflective assessment of the perceived value, relevance, and significance of statistical outputs and analytical decisions. It focuses on how statistical insights contribute to conceptual understanding, practical application, or meaningful learning, rather than just technical correctness. This form of judgement encourages learners to consider the contextual significance of their findings. Without it, statistical learning risks becoming overly mechanical, devoid of the critical reflection and creative interpretation necessary for deeper insight and academic engagement.

Dynamic evaluative judgement captures the reflective monitoring of the entire analytical process—assessing the appropriateness, accuracy, and improvement of each procedural step. It foregrounds the learner’s ability to adapt, revise, and refine their approach at every stage of the analysis. Rather than treating data analysis as a fixed sequence, dynamic evaluative judgement views it as an iterative process of learning and methodological development. Without this capacity, students may follow procedures uncritically, missing key opportunities for correction, optimisation, and deeper understanding.

By redefining these three types of evaluative judgement for statistical contexts, this study seeks to illuminate how students can develop a more comprehensive and critical approach to quantitative inquiry, an essential competence for thriving in today’s evidence-driven academic landscape.

### 2.3. Intersections Between Evaluative Judgement, Peer Feedback, and ChatGPT Feedback

As higher education environments become more complex and data-driven, students are increasingly required to navigate diverse sources of feedback and exercise discernment in their learning processes. This growing demand has prompted scholarly interest in how different feedback modalities, particularly peer feedback and ChatGPT feedback, contribute to the cultivation of evaluative judgement.

“Peer-assisted learning” refers to a process in which individuals from similar social groups, who are not professional educators, support one another’s learning and, in doing so, enhance their own understanding (Topping & Ehly, 1998). Within this concept, peer feedback has emerged as a structured and intentional strategy through which learners evaluate and respond to each other’s work. It has been widely recognised as an effective means of developing evaluative judgement, defined as the capacity to appraise the quality of one’s own work and that of others (Tai et al., 2018). Through the act of giving and receiving feedback, students engage with assessment criteria, articulate their reasoning, and negotiate shared understandings of quality. However, peer feedback is not uniformly effective. Jonsson’s (2013) review of 103 empirical studies identified several obstacles that hinder students’ engagement with feedback. Learners may disregard feedback if they find it irrelevant, vague or impersonal, excessively authoritative, difficult to apply, or laden with unfamiliar academic language. These challenges are particularly salient in domains such as statistical analysis, where technical complexity and limited prior knowledge can further hinder productive peer-to-peer dialogue. To improve the efficacy of peer feedback in such contexts, pedagogical interventions that promote sense making have shown promise. Sense-making support, through structured guidance and scaffolding, can help learners actively process, interpret, and apply feedback, thereby reducing misunderstandings and enhancing the utility of peer feedback (Wichmann et al., 2018). Specifically, these supports encourage students to reflect on feedback in relation to their planning, monitoring, and evaluation processes, enabling deeper engagement and more informed revisions.

The emergence of generative AI tools such as ChatGPT has introduced a new dimension to feedback practices, prompting questions about how these technologies might complement or extend traditional forms of peer feedback. AI-generated feedback offers several notable affordances: it is immediate, consistent, and accessible on demand. However, despite its potential, ChatGPT feedback is constrained by several critical limitations. Unlike peer feedback, which is embedded in interpersonal dialogue, contextual sensitivity, and mutual understanding, ChatGPT responses tend to be generic, occasionally vague, and often lack the specificity required for targeted improvement (Su et al., 2023). Moreover, AI-generated content can appear coherent and authoritative while containing “hallucination” (Essien et al., 2024; Rudolph et al., 2023). Furthermore, the performance of generative AI is intrinsically tied to the quality of its training data. When this data contains biases, the resulting outputs—including feedback on students’ work—may reflect and perpetuate those biases, leading to responses that are inappropriate, misleading, or unjust (Rawas, 2024).

This raises a significant concern. As Bearman and Ajjawi (2023) point out, how can learners evaluate the credibility of AI-generated feedback when the reasoning behind it remains opaque? To address this challenge, they introduce the concept of “epistemic doubt”—a cognitive and affective state marked by uncertainty and discomfort. This concept is especially pertinent in AI-supported learning environments, where students must engage critically with outputs that may appear authoritative yet are potentially ambiguous, biased, or flawed. Developing the capacity to hold such doubt is essential for navigating the inherent uncertainties of interacting with generative AI.

Furthermore, as Bearman et al. (2024) aptly note, “AI has widened the gap between our capability to produce work, and our capability to evaluate the quality of that work. It is easier than ever to produce something that has the superficial appearance of quality—but knowing if it is good enough for any given purpose requires expertise” (p. 903). This observation underscores the urgent need to support students in developing the evaluative skills necessary to navigate the ambiguity of AI outputs. To address this challenge, Bearman et al. (2024) further argue that generative AI can support the development of evaluative judgement in three key ways. First, evaluative judgement can be cultivated through the critical assessment of generative AI outputs. Given that generative AI often produces responses that are syntactically fluent and superficially convincing, there is a significant risk that students with limited subject knowledge may accept such outputs uncritically. Developing evaluative judgement in this context requires students to engage in sustained scrutiny of AI-generated content—questioning its validity, cross-referencing with other sources, and aligning it with disciplinary standards. Second, by enabling students to assess AI processes, a central challenge in working with generative AI lies in crafting precise, contextually appropriate prompts—an iterative skill that significantly influences the quality of the AI’s output. Through repeated engagement, students learn not only to refine their prompting strategies but also to evaluate whether their inputs are sufficient to elicit meaningful and accurate responses. Beyond these technical considerations, students are also encouraged to reflect on more nuanced process-related questions—particularly ethical ones. They must consider when it is appropriate to stop using AI tools altogether, especially in situations where continued reliance might undermine academic integrity, independent thinking, or the authenticity of their work. Third, generative AI enables a direct comparison between students’ evaluative judgements and those generated by the system itself. When AI tools provide assessments of student work, they create an opportunity for learners to engage in a form of reciprocal evaluation. Students are not only the recipients of feedback but also active participants in appraising its validity by comparing the AI’s assessments with their own. This process fosters critical reflection on the accuracy, consistency, and rigour of their evaluative decisions, encouraging students to question whether their judgements align with established standards and to identify gaps in their reasoning.

A critical challenge in leveraging AI feedback for learning lies in how students engage with it, whether as a tool for deepened reflection or as a shortcut to quick answers. Zhan and Yan (2025) argue that the capacity to make meaningful comparisons is central to integrating AI feedback into evaluative judgement. Building on Nicol’s (2021) work, they identify comparison as a key mechanism by which learners align external feedback—whether from AI, peers, teachers, or self-assessment—with evolving internal standards of quality. When students iteratively compare diverse sources of feedback, they are better positioned to construct a nuanced understanding of what constitutes strong academic work. However, this developmental process hinges on students’ ability to generate precise prompts, critically appraise AI-generated responses, and flexibly adapt their strategies for feedback engagement (Bearman et al., 2024; Zhan et al., 2025). Despite its potential, many students interact with ChatGPT in a cognitively passive manner, accepting responses at face value rather than interrogating their validity or relevance. Zhan and Yan (2025) caution that such uncritical reliance on AI risks displacing essential metacognitive processes. This tendency reflects what Fan et al. (2025) term “metacognitive laziness”—a pattern where learners bypass reflective analysis in favour of instant solutions. While cognitively efficient, this habitual offloading of thinking to AI may undermine students’ long-term capacity for deliberate reasoning, evaluative precision, and independent academic judgement.

Despite growing interest in the role of feedback in higher education, an important gap remains in understanding how different feedback modalities—namely, peer feedback and AI-generated feedback—compare in shaping students’ evaluative judgement. While existing studies have explored each feedback mode in isolation, few have conducted systematic, comparative analyses of their respective affordances and limitations. Even less attention has been paid to how these feedback forms cultivate hard evaluative judgement (i.e., navigating accuracy and accountability); soft evaluative judgement (i.e., cultivating reflective and interpretive depth); and dynamic evaluative judgement (i.e., reflecting on process, not just product), particularly in cognitively demanding areas such as statistical analysis. This gap has significant pedagogical implications. Without a nuanced understanding of how different feedback sources foster these layered judgement capacities, educators risk either underutilising emerging AI tools or over-relying on them at the expense of collaborative learning. Addressing this gap is crucial for designing instructional strategies that treat peer feedback and AI feedback not as interchangeable supports but as complementary mechanisms in cultivating evaluative judgement as a sustainable, transferable academic skill. To this end, the present study investigates how doctoral students in language and education disciplines develop evaluative judgement in the context of statistical analysis. Specifically, this study is guided by the following research question: how do peer feedback and ChatGPT feedback, respectively, support students in developing hard, soft, and dynamic evaluative judgements in statistical analysis?

## 3. Methods

### 3.1. Research Context and Participants

This study investigated the pedagogical potential of ChatGPT-generated feedback and peer-based feedback in supporting the development of evaluative judgement among doctoral students enrolled in a 14-week statistical analysis course at a research-intensive university in Malaysia. The course was embedded within a Higher Degree by Research (HDR) programme and specifically tailored for students in language and education disciplines, most of whom entered the programme with little to no prior experience in quantitative methods or statistical reasoning. The course aimed to build foundational competence in statistical thinking while also encouraging critical engagement with data-driven research practices.

Structurally, the course was divided into two phases. The first half (Weeks 1–7) introduced students to essential statistical concepts, such as descriptive statistics, probability theory, and correlation analysis—topics typically covered in undergraduate statistics courses. The second half (Weeks 8–14) advanced into more complex procedures, including regression modelling and multivariate analysis, with an emphasis on interpreting and applying these methods to real-world educational research contexts. Throughout the course, students participated in guided tutorials and were required to work with open-access datasets, fostering hands-on engagement with software tools such as SPSS and encouraging the practical application of theoretical knowledge.

As part of their mid-term assessment, students were tasked with conducting a step-by-step correlation analysis using an open-access dataset. This involved choosing between Pearson Spearman correlations, formulating a relevant null hypothesis, demonstrating the correct use of SPSS for correlation testing, interpreting the statistical output, and presenting the findings in the format of an academic research report. This task required students to carry out a complete correlation analysis using an open-access dataset. Specifically, they were expected to determine the appropriate statistical test—choosing between Pearson and Spearman correlation methods—based on the nature of their data. Once the appropriate test was selected, students were required to formulate a relevant null hypothesis, clearly articulating the expected relationship (or lack thereof) between the variables. They then had to demonstrate the correct use of SPSS to perform the correlation analysis, including accurately navigating the software interface, selecting relevant variables, and applying appropriate statistical settings. Following the analysis, students were expected to interpret the statistical output, such as correlation coefficients and significance values, with attention to both statistical accuracy and contextual relevance. Finally, they were required to present their findings in the format of an academic research report, incorporating methodological justification, analytic interpretation, and appropriately structured reporting in line with scholarly conventions.

To examine the impact of different feedback modalities on students’ evaluative development, the 32 enrolled doctoral students either received feedback generated by ChatGPT or structured peer feedback from their classmates. This number reflects the total cohort of doctoral students available at the time, within a research environment that was under the researchers’ control. Following the assignment, all students submitted written reflections on their feedback experiences, allowing for an initial comparison of their perceptions and responses to the two feedback modalities. To gain a deeper understanding of these feedback experiences, six students were purposively selected for follow-up semi-structured interviews. The selection criteria focused on students who demonstrated active engagement in the feedback process, as evidenced by video recordings of peer feedback sessions or screenshots of their interactions with ChatGPT. The purposive sampling approach included both typical and information-rich cases to reflect meaningful variation without relying on extreme outliers. This approach ensured balanced representation across feedback modalities, enhancing the dataset’s credibility. Despite the small sample size, the diversity and relevance of participants’ experiences allowed for the identification of recurring patterns and key contrasts, supporting both analytical depth and a robust understanding of how different feedback modalities shaped students’ evaluative development.

The selected participants included three students who received ChatGPT feedback (Mary, John, and Tina) and three students from the same peer feedback group (Olivia, Stella, and Viki). Interview data revealed that each type of feedback contributed differently to the development of evaluative judgement across three interrelated dimensions: hard, soft, and dynamic. These findings offer nuanced insights into how AI and peer feedback shape students’ ability to critically assess and justify statistical decisions within a research-based learning environment.

### 3.2. Data Collection

**Figure 1:**
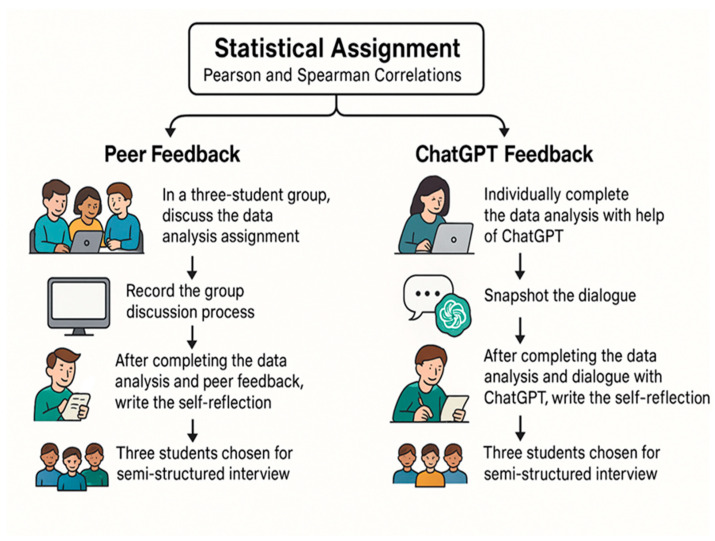
Assignment workflow comparing peer feedback and ChatGPT feedback for statistical analysis.

This study adopted a multi-source, task-integrated data collection strategy to capture the complex ways in which different feedback mechanisms—peer-based and AI-generated—influence doctoral students’ evaluative judgement in statistical analysis, as illustrated in Figure 1. The approach was designed to triangulate perspectives from both performance-based and reflective data, thereby enabling a holistic understanding of how students engaged with feedback throughout the mid-term assignment process.

Data were drawn from five complementary sources. For the mid-term assignment, all 32 enrolled doctoral students first completed the feedback procedure, followed by the post-implementation survey. These surveys gathered students’ self-reported perceptions of the feedback they received, focusing on its perceived accuracy, relevance, and its influence on their analytical thinking. The responses provided the researchers with a general understanding of how students perceived different feedback modalities. Second, six participants—three from each feedback group—were purposively selected for semi-structured interviews. These interviews employed a task-based interview method (Mejía-Ramos & Weber, 2020), which is particularly well-suited to eliciting students’ reasoning and cognitive processes in response to specific learning tasks. Interviewees were asked to revisit their statistical analysis and respond to open-ended prompts such as the following: “Do you think the peer feedback or ChatGPT feedback was accurate for evaluating your data analysis?” “How important was the feedback for your understanding?” and “In what ways did the feedback guide your step-by-step analysis?” These interviews generated rich qualitative insights into how students internalised and responded to different forms of feedback.

In addition to surveys and interviews, this study incorporated authentic records of student interaction with feedback sources. For the peer feedback group, students engaged in small-group discussions to collaboratively analyse their assigned task. These group sessions were video recorded to capture the flow of dialogue, negotiation of interpretations, and emergence of shared understanding. This audiovisual data provided a window into the social and discursive processes through which evaluative judgement was co-constructed. In the ChatGPT feedback group, students worked independently and were instructed to take screenshots of their interactions with the AI while completing the assignment. These dialogue snapshots served as evidence of how students framed questions, interpreted AI-generated responses, and integrated feedback into their workflow.

All students also submitted their completed mid-term assignments, which required them to determine the appropriate correlation test (Pearson or Spearman), formulate a null hypothesis, perform the analysis using SPSS, Version 30 (Statistical Package for the Social Sciences, IBM, Chicago, IL, USA), interpret the statistical output, and report their findings in the format of an academic paper. These written artefacts functioned as tangible representations of students’ applied evaluative skills and provided a critical basis for comparing learning outcomes between the two feedback conditions.

This study was conducted in compliance with institutional ethical guidelines approved by the university’s research ethics committee. All participants were thoroughly briefed on this study’s aims, procedures, and their rights and were provided written informed consent. Measures were taken to protect participants’ anonymity and confidentiality, including the use of pseudonyms (e.g., Mary, John, and Viki) in data analysis and reporting. Participation in interviews and the submission of peer discussion recordings or ChatGPT dialogues was entirely voluntary, and participants were free to withdraw at any time without consequence. All digital materials were securely stored on encrypted servers and password-protected devices, ensuring that access remained restricted to authorised members of the research team.

### 3.3. Data Analysis

Our analytical approach was informed by Nelson’s (2018) evaluative judgement framework, which conceptualises evaluative judgement as comprising three interrelated dimensions: hard, soft, and dynamic, as explained above. To examine how these dimensions emerged in participants’ learning experiences, we employed a thematic analysis procedure that combined both inductive and deductive coding techniques (Fereday & Muir-Cochrane, 2006). This dual strategy enabled us to capture emergent insights from the data while also aligning them with established theoretical constructs. The analysis was conducted using NVivo, version 1.5.1 (Lumivero, Denver, CO, USA) and focused on exploring how HDR doctoral students in language and education disciplines developed evaluative judgement in the context of statistical analysis.

The analysis began with a close reading of all post-implementation survey responses and interview transcripts by the principal researcher, who used open coding to identify salient ideas, expressions, and recurring evaluative patterns in the data. These initial codes informed the development of a preliminary coding manual, which was then collaboratively reviewed and refined by the research team. To enhance credibility and coding reliability, a portion of the data was independently coded by three researchers, with discrepancies resolved through discussion and consensus. Using the validated codebook, the dataset was systematically coded and summarised to identify emerging patterns in how students engaged with feedback and exercised evaluative judgement. At this stage, the team applied a deductive coding template derived from Nelson’s (2018) framework to classify instances of judgement as hard, soft, or dynamic. Meanwhile, we remained open to additional codes that extended or challenged the framework, allowing for both data-driven and theory-informed insights. In the next stage of analysis, the research team collaboratively organised the codes into coherent thematic clusters through a series of iterative discussions. This process allowed us to map the connections between students’ reflections and the three types of evaluative judgement—hard, soft, and dynamic. We paid particular attention to episodes that illustrated how students navigated issues of accuracy and accountability, cultivated reflective and interpretive depth, and engaged in process-oriented reflection beyond the final product. To ensure conceptual clarity, overlapping or ambiguous codes were revisited and refined. To enhance the trustworthiness of our findings, we triangulated the emergent themes across multiple data sources, including post-implementation surveys, interview transcripts, video recordings of the peer feedback process, snapshots of students’ dialogues with ChatGPT, and their statistical assignment results. This multi-source triangulation enabled us to cross-validate interpretations and assess the extent to which the themes aligned with the guiding research questions and the theoretical framework. Each theme was substantiated with illustrative quotes, while disconfirming evidence was actively considered to refine our interpretations. This final analytic phase ensured that the themes were both empirically credible and theoretically coherent.

## 4. Findings

### 4.1. Hard Evaluative Judgement

In the context of statistical analysis, hard evaluative judgement is anchored in the pursuit of accuracy, both in terms of the correctness of computational outputs and the appropriateness of the analytical methods employed. It requires students to move beyond superficial error detection, such as identifying data entry mistakes or miscalculations, and to engage in deeper scrutiny of statistical choices, including the selection, application, and interpretation of tests. This form of judgement plays a critical role in ensuring methodological rigour in research tasks. Table 2 compares the development of hard evaluative judgement in statistical data analysis under the influence of ChatGPT feedback and peer feedback.

Among students who received ChatGPT feedback, the development of hard evaluative judgement was shaped by their need to critically engage with AI-generated outputs. Tina, for example, reported a relatively smooth experience when the statistical tasks were straightforward, acknowledging that ChatGPT’s responses were generally accurate under such conditions. However, her process also highlighted the importance of verifying that the AI’s suggestions aligned with the context and objectives of the analysis. She became increasingly aware that the quality of the prompts she provided could significantly influence the responses she received. This prompted her to approach ChatGPT’s outputs with a more critical lens, recognising that even correct-looking answers could be based on flawed assumptions if the initial prompt lacked clarity or precision. Furthermore, John, another student in the ChatGPT group, also demonstrated the use of hard evaluative judgement through a process of careful comparison between his own results and those provided by the AI. Rather than relying passively on ChatGPT’s feedback, he made a point of scrutinising the logic and reasoning behind the outputs. His approach reflected a growing capacity to evaluate not only whether the statistical values were correct but also whether the analytical procedures and interpretations aligned with accepted standards in the field. This critical engagement with AI feedback marked a shift from acceptance to interrogation, underscoring the active role that students must play when using AI as a learning support tool.

In the peer feedback group, hard evaluative judgement was exercised in a different but equally important way. Stella’s experience illustrates how comparing peer responses with her own work allowed her to detect errors that had gone unnoticed during self-checking. The process of peer comparison acted as a trigger for deeper reflection, encouraging her to revisit assumptions and re-evaluate the correctness of her analysis. This kind of peer-based discrepancy identification proved to be an effective prompt for developing critical awareness. However, the collaborative nature of peer feedback also raised concerns about the reliability of the feedback itself. Viki, for instance, expressed uncertainty when her group reached a consensus on certain answers. Her reflection revealed an awareness that agreement among peers did not necessarily equate to correctness. In these instances, she felt the need to consult a more authoritative source to validate their conclusions. This sense of ‘‘epistemic doubt’’ (Bearman & Ajjawi, 2023), recognising the limits of peer authority and seeking external validation, demonstrates a key component of hard evaluative judgement in the peer feedback context.

Overall, while the two feedback modalities presented different challenges, both environments prompted students to actively exercise hard evaluative judgement. ChatGPT feedback required students to validate the procedural accuracy and interpretive soundness of algorithm-generated outputs, especially in light of prompt limitations. Peer feedback, on the other hand, encouraged critical comparison and reflexive questioning of both one’s own work and the input of others. Together, these findings illustrate how hard evaluative judgement is not simply about error detection but involves a deeper metacognitive engagement with statistical reasoning and methodological correctness.

### 4.2. Soft Evaluative Judgement

In the context of statistical analysis, soft evaluative judgement refers to a learner’s ability to assess the significance, relevance, and value of statistical findings or methods. Unlike hard evaluative judgement, which focuses on factual correctness and procedural validity, soft judgement involves reflective questions such as the following: “What does this result contribute?”, “Does this approach enhance interpretive depth?”, or “How useful is this insight for understanding the bigger picture?” This form of judgement plays an important role in shaping analytical perspectives and fostering a deeper engagement with the meaning of data beyond technical correctness. Table 3 compares the development of soft evaluative judgement in statistical data analysis across the two feedback mechanisms.

Students using ChatGPT feedback demonstrated varying degrees of soft evaluative judgement development. For Mary, ChatGPT’s immediacy and accessibility served as a reliable entry point for exploring ideas. The platform’s 24/7 availability helped her feel supported and empowered to pursue questions in real time, contributing to a more fluid and responsive learning process. For John, ChatGPT expanded the scope of his thinking by offering alternative ways of interpreting data—sometimes revealing patterns or perspectives he had not initially considered. This aligns with the idea that AI tools can serve as cognitive partners, prompting users to move beyond routine interpretations and consider broader analytical possibilities. However, the value of these AI-assisted insights was not accepted without hesitation. Tina’s experience highlighted the tension between intellectual stimulation and ‘‘epistemic doubt’’ (Bearman & Ajjawi, 2023). While ChatGPT occasionally offered useful directions, she questioned the reliability of its explanations, particularly when answers appeared to be fabricated or lacking transparency. Her reluctance to rely on ChatGPT for complex or high-stakes interpretation reflected a growing awareness that novelty or convenience does not always equate to conceptual soundness. Furthermore, her preference for traditional tools such as SPSS stemmed from a perceived lack of clarity about ChatGPT’s underlying logic, algorithms, or training data. This suggests that the contribution of AI to soft evaluative judgement is contingent upon the learner’s ability to discern when AI outputs enhance understanding and when they risk undermining it.

In contrast, peer feedback promoted soft evaluative judgement primarily through collaborative dialogue and mutual reflection. Olivia’s experience illustrates how peer input enriched her interpretive processes. By engaging with alternative explanations and receiving feedback that pointed out ambiguities or presentation issues, she was able to refine the significance and clarity of her statistical interpretations. This form of discursive peer engagement stimulated deeper thought about what makes findings compelling or meaningful in academic communication. Stella’s reflection further demonstrates the motivational and affective benefits of peer interaction in fostering soft evaluative judgement. Through active participation and the sense of being part of a learning community, she reported greater confidence and engagement. Meanwhile, Olivia also described moments of co-regulated learning, in which her peers recognised strong contributions and constructively resolved disagreements. Such shared decision-making processes provided opportunities to collectively appraise the value and presentation of statistical insights, reinforcing judgement as a socially co-constructed act. Nonetheless, some limitations of peer feedback also emerged. Viki, for instance, expressed reservations about trusting peer input when deeper statistical expertise was required. Her awareness that peers were similarly novice learners prompted caution in fully accepting their evaluative comments. This highlights a boundary of peer-led soft judgement: while peers can enhance motivation, awareness, and reflection, they may fall short in ensuring analytical depth without expert scaffolding. Therefore, the contribution of peer feedback to soft evaluative judgement may lie less in the precision of the feedback and more in the reflective habits and critical conversations it stimulates.

In summary, both ChatGPT feedback and peer feedback contributed to the development of soft evaluative judgement, albeit through different mechanisms. ChatGPT fostered immediacy, broadened thinking, and introduced novel perspectives, though sometimes at the expense of reliability. Peer feedback facilitated reflective dialogue, shared meaning making, and motivational support, though it was occasionally constrained by participants’ limited statistical expertise. These findings suggest that soft evaluative judgement is best cultivated in environments that balance innovation with critical dialogue and encourage students to evaluate not only what they learn, but why it matters.

### 4.3. Dynamic Evaluative Judgement

In the context of statistical analysis, dynamic evaluative judgement refers to students’ ongoing engagement with the process of inquiry—monitoring, critiquing, and adjusting each analytical step rather than focusing solely on final outcomes. This form of judgement is particularly relevant when learners must navigate ambiguity, make iterative decisions, and respond to evolving insights as they work through complex tasks. Dynamic evaluative judgement thus reflects an opportunity-driven approach to learning, where students refine their understanding by actively interacting with feedback throughout the analysis process. Table 4 presents a comparative analysis of how dynamic evaluative judgement in statistical data analysis evolved under the influence of the two distinct feedback mechanisms.

In the ChatGPT feedback condition, dynamic evaluative judgement was shaped by the platform’s structured and procedural guidance. For Mary and John, ChatGPT helped clarify research questions and suggested appropriate statistical methods while also providing step-by-step instructions for conducting procedures in SPSS. These affordances made the learning experience more navigable and scaffolded, particularly for students lacking confidence in independent statistical reasoning. Mary found the tool’s explanations of results helpful for interpreting statistical meaning, while John appreciated the precision of its instructions for software use. These experiences suggest that ChatGPT can function as a procedural anchor—supporting task navigation and encouraging students to approach analysis in a systematic, step-by-step manner.

**Figure 2:**
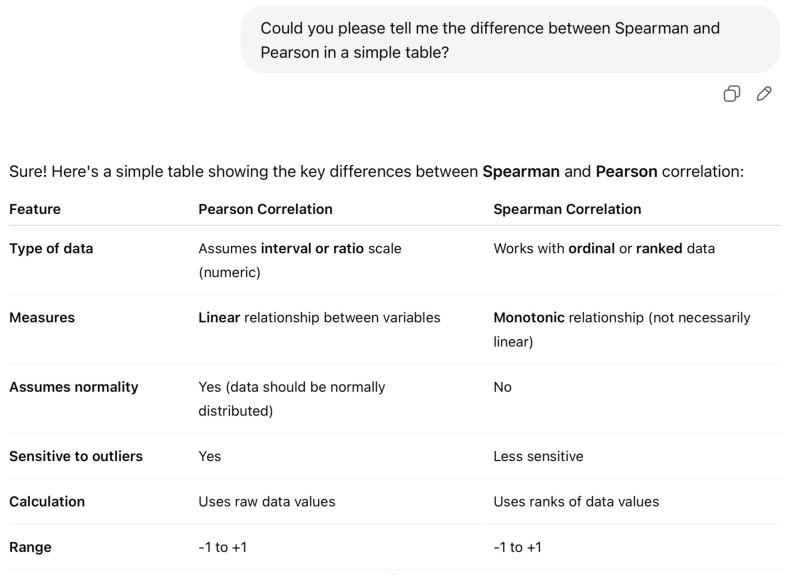
Snapshot of Mary’s dialogue with ChatGPT-4o on the differences between Spearman and Pearson correlations.

However, the effectiveness of ChatGPT in promoting dynamic evaluative judgement was constrained by its inability to detect errors or verify procedural accuracy. While the AI could articulate statistical outputs and provide general reporting guidance, it failed to flag mistakes such as selecting inappropriate variables, omitting essential steps, or misinterpreting underlying assumptions. This limitation became especially evident in Mary’s interaction with ChatGPT-4o, which highlights how students may be misled by AI’s surface fluency and authoritative tone. During a dialogue about the differences between Spearman and Pearson correlations, Mary consulted ChatGPT and received a concise, well-structured summary explaining key distinctions—such as the type of relationship each method measures, the assumptions they require, and their appropriate contexts of use. Although the response was linguistically polished and easy to follow, it lacked visual aids and omitted crucial procedural guidance—for instance, how to test whether the assumptions for Pearson’s correlation were met or how to match test choice to data types. As shown in Figure 2, Mary over-relied on the apparent clarity of the explanation without critically verifying whether her dataset met Pearson’s assumptions due to her being a novice. Consequently, she applied Pearson correlation to ordinal data—an inappropriate methodological decision that compromised the validity of her analysis. This example illustrates how superficial understanding, reinforced by the seemingly authoritative nature of AI-generated feedback, can lead learners to make decisions that are technically correct in form but fundamentally flawed in substance.

In the peer feedback setting, dynamic evaluative judgement emerged more collaboratively and dialogically. Peers played an instrumental role in prompting critical reflection on the appropriateness of statistical methods, as seen in Stella’s case. During discussions, she and her peers not only debated the use of Spearman versus Pearson correlations but also linked their decisions to lecturers’ slides and disciplinary expectations. Olivia’s experience further reinforced the procedural impact of peer interaction: when her group asked her to re-run the analysis, they identified a mistake in the data file selection—prompting a necessary correction in her workflow. This kind of real-time intervention is emblematic of dynamic evaluative judgement as an iterative process driven by social interaction and shared accountability. Moreover, peer feedback fostered a deeper understanding of statistical outputs and interpretive choices through reciprocal explanation and justification. Stella noted that her group consulted external sources and academic references to support their feedback, helping members refine their choices based on collective reasoning.

**Figure 3:**
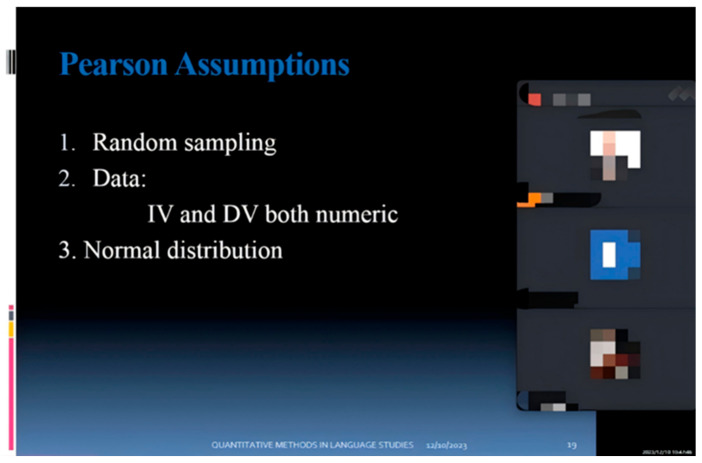
Snapshot of an online meeting: Viki, Stella, and Olivia discussing the differences between Spearman and Pearson correlations.

While this co-regulated learning environment cultivated shared responsibility and procedural accuracy, it also exposed its limitations. As Viki observed, the group sometimes reached consensus not through deep conviction but due to practical necessity. Figure 3 captures this social dimension—a snapshot of Viki, Stella, and Olivia engaged in an online meeting, discussing correlation choices in reference to lecture materials. Their negotiation reflects a blend of epistemic caution and pragmatic compromise, signalling that dynamic evaluative judgement in peer settings is both reflective and relational.

Taken together, these findings suggest that ChatGPT and peer feedback support dynamic evaluative judgement through contrasting mechanisms. ChatGPT offers structured, linear scaffolding that supports procedural navigation but requires learners to independently verify logic and detect errors. Peer feedback, on the other hand, encourages the co-construction of knowledge through dialogue, iteration, and mutual correction. Both environments reveal that dynamic evaluative judgement is not simply about following procedures but about interrogating them—continuously refining choices based on context, reasoning, and collaboration. These distinctions have important pedagogical implications. While ChatGPT can effectively support novices in managing the complexity of statistical tools, it should be paired with activities that train students to question and validate automated guidance. Peer feedback, meanwhile, benefits from structured prompts and expert oversight to mitigate the risk of superficial consensus. Ultimately, fostering dynamic evaluative judgement requires designing learning environments where reflection, dialogue, and procedural awareness are treated not as outcomes, but as integral elements of the analytical process itself.

## 5. Discussion

This study examined how peer and ChatGPT feedback support the development of hard, soft, and dynamic evaluative judgement among doctoral-level students engaging in statistical data analysis. Drawing on Nelson’s (2018) typology and recent expansions in evaluative judgement scholarship, the findings illustrate how distinct feedback mechanisms mediate learners’ engagement with analytical rigour, interpretive insight, and procedural reflection.

### 5.1. Hard Evaluative Judgement: Navigating Accuracy and Accountability

The findings underscore the centrality of hard evaluative judgement, as a foundational competence in empirical research. This form of judgement is particularly critical in doctoral-level quantitative inquiry, where incorrect methodological choices can undermine the validity of an entire research project. Yet, as Baglin et al. (2017) and Brown (2024) point out, many HDR students in language and education disciplines enter research programmes with limited statistical training, leaving them underprepared to make such technical assessments with confidence. The present study confirms this concern, revealing that, while both ChatGPT and peer feedback prompted students to engage in hard evaluative judgement, they did so through distinct mechanisms shaped by the nature of the feedback environment.

In the ChatGPT feedback setting, students frequently encountered AI-generated responses that appeared linguistically polished and procedurally correct. However, these outputs often masked deeper issues that required critical scrutiny. This highlights a central tension identified by Xing (2024) and Rawas (2024): while generative AI can produce plausible and well-structured content with a remarkable speed, such outputs are not inherently reliable—especially in technically specialised domains such as statistical analysis. Surface fluency can obscure methodological inaccuracies, misleading students who lack the evaluative skills to interrogate the underlying logic. The experiences of students like Tina and John illustrate the pedagogical significance of moving beyond the passive acceptance of AI feedback. Confronted with syntactically sound yet potentially flawed responses, they were compelled to evaluate the coherence, contextual appropriateness, and statistical validity of the information provided. These moments of cognitive dissonance point to the necessity of cultivating what Bearman and Ajjawi (2023) term “epistemic doubt”, a state of cognitive and affective unease that encourages learners to approach AI feedback with both curiosity and caution.

Importantly, epistemic doubt does not imply wholesale distrust. Rather, it promotes a stance of provisional trust, in which students tentatively engage with AI-generated feedback while remaining alert to its limitations and open to re-evaluation. This dynamic interplay between trust and scepticism fosters a more reflective and resilient engagement with AI, transforming students from passive recipients of information into active evaluators. It also aligns with what Albers (2017) identifies as core pedagogical aims in statistical education: encouraging students to assess the appropriateness of analytical choices and verify the robustness of data interpretations. In comparing their own calculations with ChatGPT’s feedback, students engaged in reflective validation—a process that both sharpened their analytical skills and deepened their understanding of statistical reasoning. Ultimately, ChatGPT’s value lay not in offering definitive answers, but in serving as a catalyst for accuracy-oriented reflection (Xing, 2024). However, this potential was only realised when students possessed, or were supported in developing, the evaluative capacities necessary to interrogate AI feedback rather than accept it uncritically.

In contrast to the individually focused and cognitively demanding engagement required by AI-generated feedback, peer feedback fostered hard evaluative judgement through a more dialogic, socially situated process. Group discussions enabled students to surface inconsistencies and identify methodological flaws in their work by juxtaposing their interpretations with those of their peers. Stella’s realisation of a previously overlooked error emerged directly through collaborative analysis, exemplifying Topping and Ehly’s (1998) conception of ‘‘peer-assisted learning’’ as a co-constructive and reciprocal endeavour. In this setting, evaluative judgement was not an isolated cognitive act but a socially mediated practice, shaped by collective efforts to justify and scrutinise analytical decisions. However, the collaborative nature of peer engagement also introduced epistemic uncertainty. When all group members lacked sufficient expertise, the risk of shared misconceptions being reinforced rather than challenged increased, a dynamic Jonsson (2013) has previously identified. In such cases, group consensus may mask rather than resolve errors. This was evident in Viki’s hesitation to accept her group’s conclusions without further verification, signalling the emergence of ‘‘epistemic doubt’’ (Bearman & Ajjawi, 2023), a critical awareness of the limitations of peer-generated knowledge and a readiness to interrogate shared assumptions. This disposition reflects what Wichmann et al. (2018) term “sense-making support”, where learners engage in reflective judgement, particularly when the credibility of peer feedback is uncertain.

It is significant to note that the development of such vigilance is not automatic; it depends on students’ capacity to critically assess the epistemic reliability of peer input. Recognising the potential fallibility of group reasoning prompts students to activate metacognitive strategies for validating information, especially when confidence in shared knowledge is low. This underscores the need to scaffold peer feedback practices with explicit criteria and strong statistical grounding (Ellis & Slade, 2023; Schwarz, 2025). The effectiveness of peer feedback in cultivating hard evaluative judgement significantly increases when students are equipped with the necessary disciplinary knowledge and pedagogical tools (Bearman et al., 2024). Without such support, peer-assisted learning may perpetuate uncertainty rather than resolve it, thereby limiting its potential as a formative space for rigorous methodological scrutiny.

### 5.2. Soft Evaluative Judgement: Cultivating Reflective and Interpretive Depth

The development of soft evaluative judgement—understood as the ability to assess the interpretive value, coherence, and contextual relevance of statistical findings—was closely tied to the extent to which feedback stimulated reflective engagement.

In cultivating soft evaluative judgement, ChatGPT functioned as both a catalyst and a constraint. For students like John, the immediacy and accessibility of AI-generated feedback opened up exploratory pathways that broadened their conceptual horizons. ChatGPT’s ability to propose alternative perspectives and unfamiliar lines of reasoning supported more flexible, expansive engagement with statistical content. Such moments of intellectual curiosity reflect what Bearman et al. (2024) describe as the role of generative AI as a “partner”, extending students’ interpretive reach beyond the boundaries of prior knowledge. Importantly, this form of value-adding support does not rest solely on the accuracy of AI outputs, but rather on their capacity to provoke new insights, foster exploratory thinking, and scaffold emerging evaluative capabilities. Used well, AI tools can help students learn not just what to think, but how to think more broadly within a domain.

However, the benefits of this partnership were unevenly realised. Tina’s preference for SPSS, which she regarded as more transparent and reliable, highlights persistent concerns about ChatGPT’s lack of algorithmic transparency and the potential impact this opacity may have on the trustworthiness and interpretability of AI-generated results. Unlike conventional software grounded in traceable logic and fixed algorithms, generative AI operates via opaque, probabilistic processes and is trained on unverifiable data sources (Essien et al., 2024). This epistemic opacity contributes to a key pedagogical risk: “hallucination” (Rudolph et al., 2023), which can obscure rather than clarify understanding. When students cannot distinguish between plausible insight and fabricated explanation, the development of evaluative judgement is undermined. As Nelson (2018) emphasises, evaluative judgement is not just about selecting reasonable answers but also articulating why one interpretation is more valuable or defensible than another. Thus, the pedagogical potential of AI in supporting soft evaluative judgement ultimately depends not only on its generative capabilities, but also on students’ critical engagement, epistemic vigilance, and capacity to interrogate the validity and relevance of what AI offers.

In contrast to the individualised, tool-mediated engagement of AI feedback, peer feedback played a pivotal role in cultivating soft evaluative judgement through social interaction, mutual reflection, and collaborative inquiry. For students such as Olivia and Stella, peer exchanges offered more than opportunities to compare answers; they created a dialogic space for questioning assumptions, clarifying reasoning, and considering alternative interpretations of statistical findings. These conversations encouraged students to move beyond superficial understanding toward deeper, conceptual engagement with analytical processes. By articulating their reasoning and responding to others’ interpretations, students engaged in the kind of deliberative, effortful thinking that Joughin (2018) associates with the analytical mode of dual-process theory, a mode essential for developing sound evaluative judgement. Moreover, the social nature of peer feedback contributed to learners’ epistemic motivation and confidence. For students with limited prior experience in statistics, peer discussions helped demystify the subject by framing it as a shared learning challenge rather than an isolating technical task. This collaborative framing supported learning motivation, particularly among students in non-STEM fields, by fostering a sense of belonging and mutual responsibility for intellectual progress. Through this process, students began to view statistical analysis not merely as a mechanical skill to master, but as a dynamic form of inquiry that could be approached collectively. Crucially, peer feedback also fostered co-regulated learning, a mode of shared cognitive and emotional regulation wherein learners support one another’s meaning making and problem solving. The iterative process of explaining, challenging, and revising interpretations enabled students to internalise evaluative criteria and co-construct standards of quality. This aligns with Goodyear and Markauskaite’s (2018) conceptualisation of evaluative judgement as an epistemic capability—a generative skill that enables individuals to navigate complexity, adapt standards, and make principled decisions in evolving professional contexts. As they argue, in fast-changing environments, learners must develop epistemic resourcefulness: the ability to formulate novel yet defensible judgements, rather than rely on fixed rules or static models.

Nevertheless, the peer feedback process was not without limitations. In contexts where statistical expertise was limited, peer insights sometimes lacked sufficient depth or rigour. Viki’s cautious stance toward her peers’ interpretations, particularly when they diverged from statistical norms, points to the delicate balance between motivational support and epistemic authority. While peer dialogue may enhance reflective engagement, its capacity to foster conceptual accuracy depends heavily on disciplinary scaffolding and structured guidance. As noted by Chen et al. (2022), peer feedback is most effective when supported by explicit criteria and expert oversight. This tension between collaborative support and epistemic doubt reflects what Dall’Alba (2018) describes as an ontological dimension of learning: students are not merely acquiring skills or knowledge but are also learning to become researchers—individuals capable of exercising critical judgement within complex and uncertain knowledge environments. As such, peer feedback serves not only to correct immediate errors but to foster a disposition of methodological scepticism and intellectual accountability. This is particularly vital in non-STEM research contexts, where statistical reasoning may be underemphasised but remains essential to producing robust empirical work.

### 5.3. Dynamic Evaluative Judgement: Reflecting on Process, Not Just Product

Dynamic evaluative judgement emerged in this study as a key point of differentiation between feedback modalities, underscoring its centrality in tasks, such as statistical analysis, that demand procedural reasoning and continuous interpretive refinement (Albers, 2017). As Nelson (2018) suggests, such tasks require learners not only to reach correct outcomes but to engage in recursive decision making across successive stages of analysis. Rather than treating evaluative judgement as a discrete skill, recent scholarship views it as a holistic, evolving capacity grounded in disciplinary engagement (Luo & Chan, 2022a, 2022b). Within this perspective, the ability to make meaningful comparisons becomes a central developmental mechanism. Zhan and Yan (2025) emphasise that iterative comparison, between external feedback and internal standards, and across sources such as AI, peers, instructors, and the self, is fundamental to cultivating evaluative judgement. Students who engage in such comparative reasoning are more likely to develop a nuanced and context-sensitive understanding of quality.

The ChatGPT feedback environment supported dynamic evaluative judgement by offering step-by-step procedural scaffolding, which helped students navigate statistical software and clarify analytical sequences. For students like Mary and John, this structured guidance not only facilitated technical fluency but also enabled them to link specific analytical procedures to broader research objectives. These findings align with Ellis and Slade’s (2023) and Schwarz’s (2025) framing of AI as a cognitive amplifier—capable of reducing technical barriers and enhancing learners’ engagement in complex, data-intensive tasks. However, the benefits of ChatGPT feedback were not uniformly realised across all students. A key limitation emerged when learners treated the AI as an authoritative source rather than as a partner in critical inquiry. When students lack the capacity to assess the accuracy, relevance, or methodological soundness of AI-generated content, they are particularly vulnerable to accepting superficially convincing but flawed outputs (Bearman et al., 2024). Mary’s experience exemplifies this challenge, highlighting how over-reliance on AI without adequate evaluative judgement can lead to misinterpretation and procedural errors. Her case reflects a broader concern identified by Zhan et al. (2025), who argue that AI-enhanced learning environments must be purposefully designed to support comparison, iterative reasoning, and critical self-evaluation. In the absence of such scaffolding, students may become passive recipients of procedural guidance rather than active participants in the development of their analytical thinking.

In contrast, peer feedback fostered dynamic evaluative judgement through socially mediated inquiry and co-regulated reasoning. Collaborative dialogue required students not only to justify their own analytical decisions but also to engage critically with alternative perspectives and revise their approaches in response to peer input. This dynamic was clearly illustrated in Stella and Olivia’s exchanges, where the discussion around correlation test selection drew upon lecture slides, disciplinary conventions, and personal interpretations. These interactions reflect Nicol’s (2021) emphasis on comparison and negotiation as key mechanisms through which students internalise quality standards and sharpen their evaluative capacities. Furthermore, this process aligns with Ajjawi and Bearman’s (2018) sociomaterial perspective, which frames evaluative judgement as not solely an individual cognitive act but as an emergent and situated performance shaped by interactions among learners, tools, and contextual affordances. Their core argument is that a standard is neither a fixed reflection of objective truth nor merely a subjective interpretation of a culturally constructed artefact. Rather, it is enacted and negotiated through ongoing engagement with materials, peers, and disciplinary norms. What makes a sociomaterial view unique is that the ‘‘ultimate aim shouldn’t be seeking alignment with the teacher’s view, but a more dynamic perspective of standards’’ (Ajjawi & Bearman, 2018, p. 43). In the context of statistical analysis, this means that students’ evaluative judgements develop not in isolation, but through active participation in shared reasoning practices, where meanings are co-constructed, standards are dynamically interpreted, and decisions are continuously adjusted.

Yet, peer feedback was not without its challenges. As Viki’s account demonstrates, group consensus was at times shaped more by pragmatic concerns, such as time limitations or the desire to avoid interpersonal conflict (Jonsson, 2013), than by genuine analytical agreement. This highlights a persistent tension in collaborative learning environments: although peer interaction can facilitate distributed reasoning and shared insight, it also carries the risk of epistemic compromise, where students acquiesce to dominant views without critically interrogating the basis of group decisions. Such dynamics can dilute the rigour of evaluative processes, undermining the very judgement that collaborative learning aims to develop. The cultivation of dynamic evaluative judgement, therefore, requires more than participation in group dialogue; it demands that students actively navigate interpersonal complexities, question prevailing assumptions, and uphold methodological integrity under social pressure. As Luo and Chan (2022a, 2022b) contend, evaluative judgement is not a generic, transferable skill to be taught in isolation—it is a deeply situated, epistemologically informed practice shaped by learners’ disciplinary identities and values. In the context of statistical education for HDR students, this form of judgement is not optional or peripheral. It is essential to developing researchers who can critically engage with complexity, challenge normative assumptions, and contribute responsibly to knowledge production in an increasingly AI-mediated academic environment.

## 6. Implications

The findings of this study offer a number of implications for the design and implementation of learning environments aimed at cultivating doctoral students’ evaluative judgement in statistical analysis. By highlighting the complementary roles of peer and ChatGPT feedback in supporting hard, soft, and dynamic evaluative judgement, our research suggests actionable insights for students, researchers, teachers, curriculum designers, and university policy makers.

This study underscores the importance of students developing epistemic agency, that is, the ability to question, interpret, and justify methodological choices rather than merely reproducing them. ChatGPT can serve as a valuable tool for exploring analytical options and clarifying procedural steps but only when students engage with its feedback critically, not passively. Peer dialogue, meanwhile, supports students in articulating, defending, and refining their reasoning through collaborative inquiry. HDR students must learn to navigate between these two feedback modalities: using AI to scaffold independent reflection while relying on peer interaction to test and deepen their interpretations. Developing a disposition of epistemic doubt, as this study reveals, enables students to critically evaluate both AI and human input, thus strengthening their judgement across varied contexts.

This study contributes to a growing body of work that reconceptualises evaluative judgement as a situated, dialogic, and socio-technical practice. By applying Nelson’s (2018) tripartite framework in an AI-integrated learning environment, it offers a nuanced account of how feedback mechanisms differently activate the cognitive, interpretive, and procedural components of judgement. Future research can build on this foundation by investigating how various configurations of AI–human interaction—such as ChatGPT combined with peer and supervisor feedback—shape the development of evaluative judgement over time. To examine whether this combination fully leverages the strengths of each modality or introduces new drawbacks, it is recommended that future studies include three experimental groups (ChatGPT feedback, peer feedback, and ChatGPT plus peer feedback) to enable a more comprehensive comparison. Moreover, methodologically, the triangulated analysis across surveys, interviews, task artefacts, and feedback traces offers a replicable model for studying evaluative processes in authentic academic settings.

Educators designing HDR methods or statistics courses might need to move beyond simply delivering statistical knowledge and focus instead on scaffolding evaluative judgement. This includes providing opportunities for students to compare outputs across feedback sources (AI, peer, supervisor, and self), engage in critical dialogue, and apply explicit evaluative criteria. The findings show that neither peer nor ChatGPT feedback alone is sufficient; rather, their integration—alongside guided instruction—is crucial for promoting reflective and critical engagement. To support this, course design should embed structured activities that prompt students to justify their methodological choices, question underlying assumptions, and trace their reasoning processes. Such practices help cultivate a culture of inquiry and enhance students’ epistemic agency. Given the challenges associated with the quality and reliability of peer feedback, Darvishi et al. (2022) propose integrating AI and learning analytics to support more trustworthy peer-learning environments. Incorporating spot-checking mechanisms can further assist instructors in monitoring feedback quality and intervening when necessary. Instructional design should also anticipate the limitations of both feedback modalities. Peer feedback tasks should be scaffolded with expert input to mitigate the risk of reinforcing shared misconceptions. Similarly, AI-supported tasks should be paired with prompt guidance that encourages students to critically evaluate, validate, and cross-check AI-generated outputs. Together, these strategies can help students develop the evaluative judgement needed for rigorous and independent research in increasingly complex, data-driven academic contexts.

At the institutional level, the effective integration of both peer feedback and generative AI tools such as ChatGPT into research education necessitates thoughtful and future-oriented policy design. Rather than positioning ChatGPT as either a threat to academic integrity or a catch-all solution, universities should focus on promoting AI literacy as a core element of doctoral training. This entails equipping students with the critical competencies needed to interpret AI-generated feedback, evaluate its probabilistic nature, and apply it judiciously within specific disciplinary contexts. At the same time, institutional policies must also acknowledge the pedagogical value of peer-led formative assessment in fostering academic development—particularly in domains where students’ statistical proficiency varies widely. To support this dual emphasis, institutions should encourage the design of collaborative feedback structures that integrate both human and AI-generated inputs, alongside opportunities for students to compare, question, and reflect on the strengths and limitations of each. Such reflective engagement aligns with the broader educational aim of cultivating autonomous, critically-minded researchers. Accordingly, doctoral programmes should invest in assessment frameworks and pedagogical practices that position evaluative judgement not as a fixed skill to be mastered once, but as an evolving capacity essential to scholarly identity and lifelong academic inquiry.

## 7. Limitations, Future Studies, and Conclusions

While this study offers valuable insights into the development of evaluative judgement among HDR doctoral students in statistical analysis, several limitations must be acknowledged. First, the sample was drawn from a specific cohort of students in language and education disciplines, which may limit the generalisability of the findings to other academic domains, particularly those with more established quantitative traditions. Differences in disciplinary epistemologies and feedback cultures are likely to shape how students interpret and respond to both peer and AI-generated feedback. Second, this study focused on a single point in the curriculum, capturing students’ experiences during one statistical assignment. However, evaluative judgement is a developmental construct that evolves over time. Longitudinal research tracking students’ judgement trajectories across multiple courses, tasks, or research milestones would offer richer insights into how feedback practices contribute to epistemic development throughout the doctoral journey. In particular, future studies could explore the sustained impact of AI-generated feedback and peer feedback on doctoral identity formation, research confidence, and methodological rigour. Third, although this study employed a triangulated methodology, including surveys, interviews, feedback traces, and task artefacts, the interpretation of findings inevitably reflects the theoretical assumptions and positionality of the research team. Future research could benefit from integrating student-led reflections, learning journals, or digital ethnographic approaches to capture more fully the emotional, social, and cognitive dimensions that underpin evaluative judgement from the learners’ perspectives. Furthermore, given the limited number of interview participants in the present study, future research should explore whether divergent cases exist to further assess data saturation and representativeness. Identifying and examining such cases could offer valuable insights by revealing alternative perspectives that may challenge or enrich the current findings. Finally, in light of the growing integration of generative AI in higher education, further investigation is needed into the dynamics of feedback hybridity, that is, how students navigate, interpret, and synthesise input from AI tools, peers, supervisors, and self-assessment. Comparative studies across institutions, disciplines, and feedback technologies could help identify effective configurations for fostering robust and transferable evaluative capabilities. For instance, future research could investigate the design and implementation of hybrid feedback protocols, such as having students engage with ChatGPT feedback before participating in peer feedback sessions, with instructors actively facilitating and mediating the process. A follow-up longitudinal study or an experimental design comparing different sequences and combinations of feedback (e.g., AI-first vs. peer-first) using structured prompts could offer insights into how these configurations influence students’ evaluative development over time. Such studies could also examine the specific role of instructor facilitation in guiding students’ interpretation, integration, and application of feedback. These lines of inquiry would inform the development of pedagogical strategies that support critically reflective engagement and foster epistemic agency in doctoral education.

In conclusion, this study raises an important discussion point: What should educators consider when designing peer feedback or ChatGPT feedback in statistical courses? Contributing to the growing literature on evaluative judgement, this study demonstrates how both feedback modalities can support the development of hard, soft, and dynamic evaluative judgement among HDR doctoral students engaged in statistical analysis. Drawing on Nelson’s (2018) tripartite framework, the findings reveal that each feedback modality offers distinct affordances and challenges. ChatGPT promotes procedural fluency and conceptual exploration, but its value depends on students’ critical engagement and epistemic vigilance. Peer feedback fosters interpretive depth and collaborative reasoning but requires disciplinary scaffolding to guard against epistemic drift. This study argues that evaluative judgement should not be viewed as a fixed skill or a matter of individual cognition alone, but as a socially and technologically mediated capability. Developing such judgement is essential to doctoral training in an era increasingly shaped by AI, interdisciplinary inquiry, and epistemic complexity. For students to become responsible and reflexive researchers, they must learn not only to evaluate answers but to interrogate the assumptions, standards, and sources from which those answers emerge. Creating educational environments that combine AI tools with peer-led dialogue and structured guidance can help cultivate this evaluative maturity, one that is foundational to scholarly autonomy, methodological rigour, and lifelong learning.

## Figures and Tables

**Table 1:** Comparative frameworks of evaluative judgement.

Framework/Author(s)	Theoretical Lens	Key Dimensions of Evaluative Judgement	Unique Contribution
Ajjawi and Bearman (2018)	Sociomaterial	Emerges through interplay of learners, tools, and contexts	Emphasises how judgement is situated and mediated through socio-material interactions
Dall’Alba (2018)	Epistemological and ontological	Involves both what students know/do and who they are becoming	Highlights integration of knowledge, practice, and identity in digital higher education
Joughin (2018)	Dual-process theory	Differentiates between intuitive (System 1) and analytical (System 2) modes of judgement	Introduces cognitive mechanisms underlying evaluative processes
Goodyear and Markauskaite (2018)	Epistemic capability	Judgement as capability for knowledgeable action in complex, evolving contexts	Links evaluative judgement with adaptive expertise and situated action
Luo and Chan (2022a, 2022b)	Developmental and integrative	Cognitive, affective, behavioural, and identity-related dimensions; evolves through disciplinary practice	Emphasises the holistic, non-linear development of evaluative judgement over time
Nelson (2018)	Tripartite historical/typological	Hard (accuracy), soft (value/significance), dynamic (procedural reflection)	Offers nuanced typology based on disciplinary evolution

**Table 2:** Hard evaluative judgement in statistical data analysis.

Category	Category/(Axial) Code	Example
ChatGPT feedback	High statistical accuracy for straightforward tasks	I feel the main reason it worked well for me this time is because the tasks I gave it weren’t too complex or intricate (Tina_post-implementation survey).
	Necessity of verifying ChatGPT’s statistical outputs	If my results were different from what ChatGPT suggests, I didn’t just blindly trust it—I asked why, read its analysis carefully (John_post-implementation survey).
	Dependence on prompt quality	What if I gave it the wrong prompt? Then ChatGPT would still follow my instruction and produce an answer, but it might be based on a misunderstanding from the very beginning (Tina_interview).
Peer feedback	Identifying errors overlooked in self-checking	Peer feedback alerted me that other people’s answers were different from mine, and it was difficult for me to find such mistakes on my own (Stella_post-implementation survey).
	Questioning the authority of peer feedback	Sometimes we weren’t entirely sure whether the answer we agreed on was actually correct, and we needed an authoritative expert or teacher to provide us with a more credible explanation (Viki_interview).

**Table 3:** Soft evaluative judgement in statistical data analysis.

Category	Category/(Axial) Code	Example
ChatGPT feedback	Immediate and user-friendly assistance	ChatGPT can answer my questions anytime I need—it’s so convenient. There’s no time limit (Mary_post-implementation survey).
	Broadening knowledge scope through exploration of alternatives	It sometimes gave me new insights or different ways of looking at the data—ideas I wouldn’t have thought of myself (John_interview).
	Hallucination hindering deep learning and undermining trust	The answers given by ChatGPT are often made up and not based on anything solid, so I don’t fully trust it to handle complex data analysis (Tina_interview).
	Limited contribution to rigorous statistical analysis	Because I don’t fully understand ChatGPT’s algorithms, training data, or data privacy practices, I prefer to use traditional tools like SPSS for future work (Tina_post-implementation survey).
Peer feedback	Encouraging critical reflection and in-depth understanding	Peers can suggest alternative explanations, highlight areas of ambiguity, and recommend improvements in the presentation of findings (Olivia_post-implementation survey).
	Stimulating learning motivation and engagement	This kind of interaction increased the sense of participation, built up confidence, and made my learning feel more active and engaging (Stella_interview).
	Fostering co-regulated learning and knowledge sharing	When someone made a good point, we’d recognise it right away. If there was any confusion or disagreement, we just talked it through in a friendly way (Olivia_interview).
	Limited contribution to rigorous statistical analysis	Most of the peers I know are also starting from scratch—we’re all beginners in statistics, so I can’t fully trust peer feedback when it comes to final application (Viki_interview).

**Table 4:** Dynamic evaluative judgement in statistical data analysis.

Category	Category/(Axial) Code	Example
ChatGPT feedback	Recommending appropriate statistical methods	It could help me clarify my research question making sure it’s well-defined and recommend the suitable statistical analysis approaches (Mary_post-implementation survey).
	Step-by-step guidance for statistical software use	For correlation analysis, it told me to go to Analyse > Correlate > Bivariate in SPSS—super clear (John_post-implementation survey).
	Detailed instructions for result explanation	ChatGPT explained the meaning of each table and figure, making it much easier for me to understand the results (Mary_interview).
	Support for standardised statistical reporting	ChatGPT also guided me in properly reporting the results, including highlighting the significant findings to be reported in line with academic standards (John_interview).
	Limitations in error detection and procedure verification	ChatGPT can’t check for data entry mistakes, missing values, or confirm whether appropriate statistical procedures have been correctly followed (Tina_interview).
Peer feedback	Fostering critical reflection on statistical methods	Peer feedback prompted us to carefully examine data analysis for any potential flaws or limitations, and we discussed the reasons for choosing Spearman over Pearson (Stella_interview).
	Supporting careful review of analytical processes	My peers immediately asked me to re-operate the analysis, and when I opened the data file, they pointed out to me that I had opened the wrong data file (Olivia_interview).
	Promoting deeper understanding of statistical outputs	The discussion made me realise that my interpretation of the output was not as clear or accurate as I had initially thought (Olivia_post-implementation survey).
	Providing feedback supported by multiple references	The process of providing feedback required us to consult various sources to continually justify our viewpoints, helping us develop a deeper understanding of knowledge (Stella_interview).
	Peer consensus sometimes results from practical necessity	Since others in the group thought the same way, I felt like I had to compromise temporarily. After all, this was a team assignment, it wasn’t just about my own answer anymore (Viki_interview).

## Data Availability

Original data is unavailable due to privacy and ethical restrictions.
